# Non-pharmacological interventions targeting mobility among people with advanced cancer: a systematic review

**DOI:** 10.1007/s00520-024-08767-x

**Published:** 2024-08-05

**Authors:** Carmine Petrasso, Joanne Bayly, Simona Arculeo, Megan Bowers, Stefania Costi, Lise Nottelmann, Elena Turola, Elisa Vanzulli, Matthew Maddocks

**Affiliations:** 1 Cicely Saunders Institute of Palliative Care, Policy and Rehabilitation Bessemer Road, London, SE5 9PJ UK; 2 Fondazione IRCCS Istituto Nazionale Dei Tumori, Milan, Italy; 3 Physical Medicine and Rehabilitation Unit, Azienda USL – IRCCS di Reggio Emilia, Reggio Emilia, Italy; 4 Surgical, Medical and Dental Department of Morphological Sciences, University of Modena and Reggio Emilia, Reggio Emilia, Italy; 5 Research Unit for General Practice, Aarhus University, Aarhus, Denmark; 6 The Research Unit, Department of Palliative Medicine, Bispebjerg Hospital, Copenhagen, Denmark; 7 Research and Statistics Infrastructure, Azienda USL – IRCCS di Reggio Emilia, Reggio Emilia, Italy

**Keywords:** Cancer, Non-pharmacological, Exercise, Electrotherapy, Mobility, Walking

## Abstract

**Purpose:**

To synthesise evidence evaluating non-pharmacological interventions targeting mobility among people with advanced cancer, considering the type, efficacy and contextual factors that may influence outcome.

**Methods:**

Systematic review of studies of non-pharmacological interventions in adults (≥ 18 years) with advanced (stage III-IV) cancer, and assessing mobility using clinical or patient-reported outcome measures. Searches were conducted across three electronic databases (MEDLINE, EMBASE and CINAHL) up to June 2024. Methodological quality was assessed using Joanna Briggs Institute tools and contextual factors were evaluated through the Context and Implementation of Complex Interventions framework. A narrative synthesis was conducted due to clinical heterogeneity of included studies.

**Results:**

38 studies encompassing 2,464 participants were included. The most frequent mobility outcome measure was the 6-min walk test (26/38 studies). Exercise was the most common intervention, (33 studies: 27 aerobic and resistance, 5 aerobic, 1 resistance versus aerobic training) and improvements in mobility were found in 21/33 outcomes. Electrotherapy interventions led to significant improvements in mobility in 3/5 studies. Geographical factors (e.g. distance, transport, parking requirements) potentially limited participation in 18/38 studies. A lack of ethnic diversity among populations was evident and language proficiency was an inclusion criterion in 12 studies.

**Conclusion:**

Exercise and neuromuscular electrical stimulation appear to improve mobility outcomes in advanced cancer. The evaluation of other non-pharmacological interventions targeting mobility should consider access and inclusivity, and be adaptable to the needs of this population.

**Supplementary Information:**

The online version contains supplementary material available at 10.1007/s00520-024-08767-x.

## Introduction

Cancer is one of the leading causes of global morbidity and mortality worldwide [1]. The burden posed by advanced cancer, i.e. progressive and incurable with extensive local or metastatic involvement [2], reduces functional capacity and mobility status [2, 3]. People with advanced cancer often report debilitating symptoms, physical limitations, and reduced quality of life, culminating in difficulty completing activities of daily living [4] and emotional distress for both the individual and their family [5].

Mobility status, defined as "an individual’s ability to move oneself (either independently or by using assistive devices or transportation) within environments that expand from one’s home to the neighbourhood and to regions beyond" [6], is an important but often overlooked concept [3, 7]. Declining mobility status is considered to be one of the most unpleasant symptoms that reduces quality of life in people with advanced cancer [3, 7]. Consistent negative correlations are found between the loss of mobility and worsening pain, fatigue and/or breathlessness [8], and on psychosocial well-being [9].

Individuals with advanced cancer may become deconditioned and find themselves entrapped in a vicious cycle, whereby pain, fatigue, and breathlessness restrict their mobility, consequently exacerbating these symptoms further [5]. The importance of taking proactive steps to address mobility issues throughout the cancer journey is clear. There is increasing recognition of the role of non-pharmacological interventions in comprehensive cancer management [10]. In cancer rehabilitation these interventions encompass exercise programmes, breathlessness and fatigue self-management, mindfulness-based techniques, nutritional counselling, psychosocial support and more [10, 11]. Despite evidence of benefit, staff and space constraints may slow their implementation into routine cancer care [12].

Regarding interventions that may impact on mobility in advanced cancer, previous reviews have extensively evaluated the role of exercise [8, 9, 13, 14]. These reviews conclude that exercise is safe and associated with improved physical functioning and quality of life. No review to date has evaluated the range of non-pharmacological interventions available for people with advanced cancer, focusing on mobility as a primary outcome of interest. Moreover, a consideration of the level of resources, or the contextual factors that may affect mobility interventions, such as geographical or personal factors is required. Therefore, we aimed to provide a comprehensive synthesis of evidence for non-pharmacological interventions targeting mobility in people with advanced cancer. Our objectives were to: (i) identify and evaluate the efficacy of non-pharmacological interventions in optimising mobility; (ii) evaluate the staffing time, types of settings, equipment and other resources required to deliver the interventions; and (iii) explore contextual factors that may impact on the generalisability of interventions.

Methods.

We conducted a systematic review in accordance with the Preferred Reporting Items for Systematic Reviews and Meta-Analyses (PRISMA) guidelines [15]. The protocol was registered on PROSPERO (ID: CRD42023425824).

### Inclusion and exclusion criteria

Studies of any design that evaluated non-pharmacological interventions in adults (≥ 18 years) with confirmed advanced cancer and assessed mobility using clinical or patient-reported outcome measures (PROM) were included. Non-randomised studies of interventions (NRSIs) were included to ensure a comprehensive understanding of the evidence. NRSIs offer valuable insights, balancing the rigor of randomised controlled trials (RCTs) with the contextual richness of observational studies, thereby supporting decision-making in both policy and practice [16]. Advanced cancer was defined as stages III-IV for solid tumours. For haematological cancers, due to staging difficulties, we adopted the operational definition proposed by Cheville et al., [17], wherein lymphoma was considered stage III, and myeloma and myelofibrosis syndrome were categorised as stage IV, regardless of their distribution, as these are considered systemic conditions. Moreover, we only included studies where the participant sample comprised ≥ 95% individuals with advanced cancer. This selection criterion was adopted to mitigate some clinical heterogeneity across the included studies. We excluded incomplete or unpublished studies, case reports, conference proceedings and papers not in English.

### Search strategy

A comprehensive search of electronic databases, including MEDLINE and EMBASE (via Ovid) and CINAHL (via EBSCO) was conducted (Full search strategy: Supplementary file Tables S1-3). Using Medical Subject Headings (MeSH), truncation, and Boolean operations, the search covered the inception of each database until June 2024. Reference lists of eligible articles, previous systematic reviews, and relevant guidelines were also hand-searched for additional citations.

### Selection of studies

An online systematic review manager, Rayyan, was used to handle records and remove duplicates. Eligibility criteria were initially applied to titles and abstracts and reviewed by one of three authors (CP, JB and MM). Full-text articles were retrieved from titles and abstracts of articles that met the review criteria or lacked sufficient information to determine suitability. The retrieved articles were then imported into Zotero, a reference management software, for full-text screening by CP and one or more authors (SA, MB, SC, LN, ET, EV). Disagreements in screening were resolved through discussion between CP, JB and MM.

### Data extraction and analysis

A standardised data extraction form was used to collect information on study design, methodology, intervention specifics, setting details, sample characteristics, contextual factors, mobility outcomes, and results. Data extraction was performed by CP and checked for accuracy by at least one other author (SA, MB, SC, LN, ET, EV). For our analysis, we utilised the mean scores, standard deviations, and other statistical data as provided by the original study authors. We tabulated the p-values, confidence intervals and effect sizes (Cohen's d and Glass's delta) as reported in the studies.

### Methodological quality assessment

The methodological quality of included studies was independently assessed by CP and one or more authors (SA, MB, SC, LN, ET, EV). RCTs were assessed using the Joanna Briggs Institute (JBI) RCT appraisal tool [18]. The remaining study designs were assessed with the JBI Quasi-Experimental tool [19]. The tools were not used to exclude papers but to understand the overall strengths and weaknesses of included literature.

### Contextual factors

Contextual factors were evaluated using the Context and Implementation of Complex Interventions (CICI) framework [20], offering a structured approach to complex interventions through three dimensions of context, implementation, and setting [20]. For the purposes of this review, the following contextual domains were considered: geographical, epidemiological, socio-cultural and socioeconomic. Each study was reviewed by CP and one or more authors (SA, MB, SC, LN, ET, EV), with potential contextual factors identified through discussion and understood as general themes across studies.

## Results

### Study retrieval and analysis

**Fig. 1:**
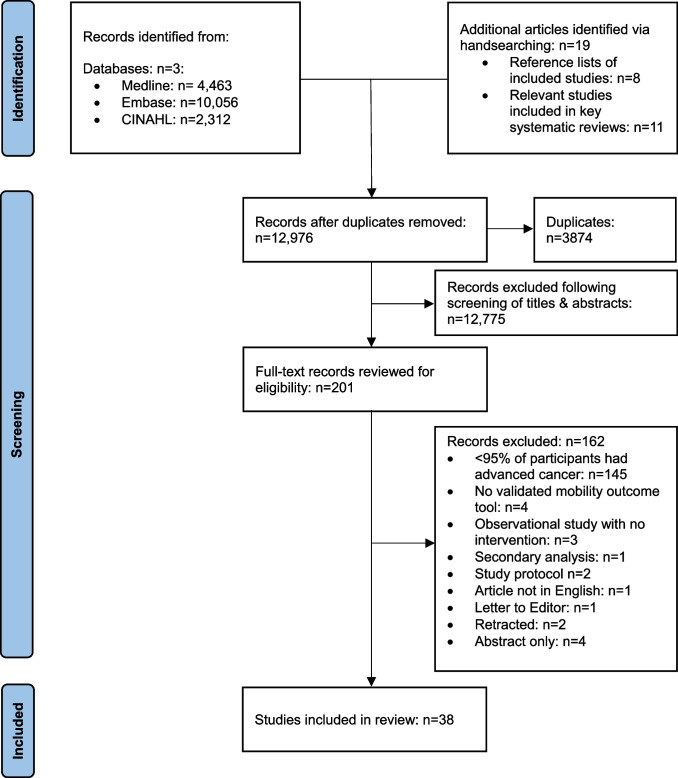
PRISMA flow diagram

The initial search yielded 16,831 articles and following the screening of titles and abstracts, 201 full-text articles were retrieved for further evaluation (Fig. 1). Subsequently, 38 articles met the eligibility criteria and were included in the review [17, 21–57]. The main reason for exclusion of full-text articles was < 95% of the study sample having advanced cancer (*n* = 145). Given the significant heterogeneity among the included studies, a meta-analysis was deemed unsuitable. Instead, a narrative synthesis was employed with data presented as tabulated summaries. Data from each article were analysed through vote counting, focusing on the statistical significance of the outcomes. Vote counting was selected due to the heterogeneity between studies and served as a pragmatic approach for conducting an exploratory analysis and to offer preliminary insights [58].

### Study characteristics

Included studies were conducted from 2009 onwards, with 10 in the USA and Canada [17, 23–25, 32, 35, 49, 51, 54, 56], 20 in Europe [21, 22, 28, 33, 34, 36–41, 44–48, 50, 52, 53, 57], six in Oceania [26, 27, 29–31, 55] and two in Asia [42, 43] (Table 1). Data from 2,464 participants were available, with individual study sample sizes ranging from 14 [55] to 344 [17]. Mean study sample size was 65. Thirty three studies included participants with solid tumours only [21–23, 25–34, 36–40, 42–52, 54–57], while five studies included solid and haematological cancers [17, 24, 35, 41, 53]. Twenty studies recruited participants with a singular type of primary cancer [23, 28–34, 36, 37, 42–46, 49–51, 55, 57], with lung cancer being the most prevalent and examined in 13 studies [23, 29, 30, 33, 34, 36, 37, 42–46, 51]. Mean study duration was 10 weeks (range 4 weeks – 9 months).

**Table 1:** Characteristics of included studies for narrative review

First author/ year/ country	Population (n)	Age Mean ± SD (years of sample)	Male (%)	Cancer type (%)				Cancer stage (%)		Functional status (% of sample)			
Randomised Controlled Trials:													
Bade, 2021, USA [23]	40	64.88 ± 8.69	25	Adenocarcinoma: 82.5				III: 27.5	IV: 72.5	ECOG 0: 17.5		ECOG 1: 82.5	
Cheville, 2013, USA [25]	66	64.65^a^	53	Colon: 48.5				IV: 100		AM-PAC CAT score between 50-75^b^			
Cheville, 2019^c^, USA [17]	344	65.6 ± 11.1	50.2	Haematological: 20.2		Prostate: 17.8		III: 6.6	IV: 93.4	AM-PAC CAT score between 53-66^b^			
Cormie, 2013, Australia [26]	20	72.15^a^	100	Prostate: 100				IV: 100		nr			
Dhillon, 2017, Australia [29]	111	64^a^	55	NSCLC: 95.5				III: 4.5	IV: 95.5	ECOG 0: 54.9	ECOG 1: 41.4		ECOG 2: 3.6
Edbrooke, 2019, Australia [30]	92	63.5^a^	55.4	Adenocarcinoma: 69.6				III: 44.6	IV: 52.2	ECOG 0: 31.5	ECOG 1: 56.5		ECOG 2: 12
Galvão, 2018, Australia [31]	57	70.1^a^	100	Prostate: 100				IV: 100		nr			
Henke, 2014, Germany [33]	44	nr	nr	NSCLC/ SCLC: nr				IIIA-IV: nr		KPS > 50^b^			
Maddocks, 2009, UK [36]	16	60^a^	56.3	Adenocarcinoma: 50				III: 31.3	IV: 68.8	ECOG 0: 25		ECOG 1: 75	
Maddocks, 2013, UK [37]	49	69^a^	57.1	Adenocarcinoma: 49				IV: 100		ECOG 0: 16.3	ECOG 1: 63.3		ECOG 2: 20.4
Mendizabal-Gallastegui, 2023, Spain [38]	90	56.8^a^	74.4	Gastrointestinal: 66.7				IV: 100		ECOG 0: 17.8		ECOG 1: 82.2	
Mikkelsen, 2022, Denmark [39]	84	71.8^a^	42.9	NSCLC: 46.4				III: 14.3	IV: 85.7	ECOG 0: 53.6	ECOG 1: 40.5		ECOG 2: 6
Oldervoll, 2011, Norway [41]	231	62.4^a^	37.7	Gastrointestinal: 31.6				III-IV: nr		KPS mean (SD): 79.4			
Rutkowska, 2019, Poland [46]	40	60.2^a^	67.5	Adenocarcinoma: 70				III: 60	IV: 40	WHO 0: 25		WHO 1: 75	
Scott, 2018, USA [49]	65	54 ± 11	0	Breast: 100				IV: 100		ECOG: 0-1^b^			
Stuecher, 2019, Germany [50]	44	67.1 ± 7.8	56.8	Colon: 52.3				III-IV: nr		ECOG: 0-2^b^			
Uster, 2018, Switzerland [52]	58	63.0 ± 10.12	69	NSLC: 27.6		Colorectal: 27.6		III: 1.7	IV: 98.3	WHO 0: 6.9	WHO 1: 60.3		WHO 2: 27.6
Yee, 2019, Australia [55]	14	62.2 ± 10.6	0	Breast: 100				IV: 100		ECOG 0: 29	ECOG 1: 57		ECOG 2: 14
Zimmer, 2018, Germany [57]	30	69.18^a^	70	Liver: 76.7				IV: 100		WHO: > 2^b^			
Non-Randomised Controlled Trials:													
Schink, 2018, Germany [47]	131	59.7^a^	56.5	Lung: 13.7		Colon: 16		III: 26	IV: 74	KPS mean (SD): 76.15			
Schink, 2020, Germany [48]	80	59.15^a^	56.1	Gastrointestinal: 61				III: 24.4	IV: 75.6	KPS mean (SD): 78.05			
Zhao, 2016, USA [56]	20	57^a^	95.1	Oropharynx: 70				III: 20	IV: 80	KPS: ≥ 90			
Randomised Comparative:													
Litterini, 2013, USA [35]	66	62.35 ± 13.49	83.3	Lung: 16.7				III-IV: nr		nr			
Randomised Crossover:													
Vanderbyl, 2017, USA [54]	36	64.9^a^	53.8	Lung: 50		Gastrointestinal: 50		III: 33.3	IV: 66.7	ECOG 0: 12.5		ECOG 1: 87.5	
Single Arm:													
Avancini, 2023, Italy [21]	12	57.66 ± 7.4	42	Pancreas: 58		Lung: 42		III: 25	IV: 75	ECOG 0: 33		ECOG 1: 67	
Avancini, 2024, Italy [22]	44	60.5 ± 10.7	43.2	Pancreas: 27.3	Breast: 18.2		Lung: 15.9	IV: 100		ECOG: 0-2^b^			
Chasen, 2013, USA [24]	116	61.64 ± 13.0	53	Head and neck: 16.4				III: 31.3	IV: 68.7	ECOG 1: 38.8	ECOG 2: 46.3		ECOG 3: 14.9
Cormie, 2014, Australia [27]	20	70.0 ± 9.8	85	Prostate: 85				IV: 100		nr			
Delrieu, 2020, France [28]	49	55 ± 10	0	Breast: 100				IV: 100		ECOG < 2^b^			
Hanson, 2023, USA [32]	22	71 ± 8	100	Prostate: 100				IV: 100		nr			
Kuehr, 2014, Germany [34]	40	60 ± 12	60	NSCLC: 100				III: 28	IV: 67	ECOG 0: 27.5	ECOG 1: 62.5		ECOG 2: 10
O’Connor, 2020, Ireland [40]	18	60 ± 9	30	Colorectal: 40				IV: 100		ECOG 2: 80		ECOG 3: 20	
Ozalevli, 2010, Turkey [42]	18	66.17 ± 7.33	83.3	SCLC: 38.9				IIIB: 16.7	IV: 83.3	KPS mean (SD): 66.11 ± 18.20			
Park, 2019, South Korea [43]	100	55.1 ± 8.7	46	Adenocarcinoma: 94				II: 5	IV: 95	ECOG 0: 13.0	ECOG 1: 83.0		ECOG 2: 4.0
Quist, 2012, Denmark [44]	29	63^a^	44.8	NSCLC: 82.6				III-IV: nr		WHO: 0-2^b^			
Quist, 2015, Denmark [45]	114	66^a^	50	NSCLC: 73.7				III-IV: nr		WHO: 0-2^b^			
Temel, 2009, USA [51]	25	68^a^	36	NSCLC: 100				IIIB: 16	IV: 84	ECOG 0: 40		ECOG 1: 60	
Van Den Dungen, 2014, The Netherlands [53]	29	54.5 ± 8.9	50	Gastrointestinal: 30.8				III-IV: nr		KPS mean (SD): 79.2 ± 9.4			

*nr* = not reported

*AM-PAC CAT* Ambulatory Post Acute Care Computer Adaptive Test, *ECOG* Eastern Cooperative Oncology Group, *KPS* Karnofsky Performance Score, *NSCLC* Non-small cell lung cancer, *SCLC* Small cell lung cancer, *SD* Standard deviation, *WHO* World Health Organisation

^a^ SD not specified for total study participants.

^b^ Baseline performance status not reported. Values presented represent participants' eligibility criteria for the study.

^c^ Cheville et al.,[17] had three trial arms. As trial arm 3 included a pharmacological element, this was excluded. Data presented pertains to arms 1 and 2 only.

### Methodological quality assessment

Across included studies, RCTs generally demonstrated good internal validity through the application of true randomisation, baseline participant similarity, and appropriate statistical analyses (Full quality assessments: Supplementary file Tables S4-5). A notable limitation was the absence of blinding for both participants and treatment providers, although this was anticipated given the inherent characteristics of the interventions. In a few cases, baseline similarity of treatment groups was unclear and there was insufficient clarity regarding the methods used to measure outcomes. In quasi-experimental studies, the hypothesised cause-and-effect relationships were easily identifiable. However, it also often remained unclear whether outcomes were measured with sufficient reliability.

### Overview of results and outcomes

Improvements (absolute or relative to control) in mobility were observed across 24 out of the 38 included studies (63.2%) [17, 21, 22, 24–28, 33–35, 38–45, 47, 48, 53–55] (Table 2). The 6 min walk test (6MWT) was most often used to evaluate mobility objectively and employed in 26 studies [21, 22, 24, 28–30, 33, 34, 38–40, 42–49, 51–57]. Mobility was assessed using accelerometers and clinical tools in six studies [28–30, 36, 43, 55]. Two studies utilised the Ambulatory Post-Acute Care (AM-PAC) tool [17, 25], a self-reported patient assessment and did not use a clinical measurement tool. Twenty two studies employed a PROM that assessed the physical functioning domain of mobility, [17, 23, 26–31, 37–40, 42, 43, 47, 48, 52, 53, 55, 56] including the European Organisation for Research and Treatment of Cancer Quality-of-Life Questionnaire Core 30 (EORTC-QLQ-C30, 15 studies) [21–23, 29, 37–40, 43, 47, 48, 52, 53, 55], Short-Form-36 (SF-36, 5 studies) [26, 27, 31, 38, 56], International Physical Activity Questionnaire (IPAQ, 3 studies) [28, 30, 55], or the EQ-5D-5L [17], Nottingham Health Profile (NHP) [42] and Physical Activity Scale for the Elderly (PASE) [56] in one study each. Of these studies, fifteen demonstrated improvements in some [26–28, 40] or all of the outcomes used to assess mobility [17, 21, 22, 38, 39, 42, 43, 47, 48, 53, 55]. However, only four of these studies [17, 22, 42, 55] reported improvements in both the measure for mobility and PROM.

**Table 2:** Intervention details, measure of mobility and results

First author/ year/ country	Intervention/ programme details	Duration of intervention and follow-up (weeks)	Prescribed intervention	Adherence to intervention	Mobility outcomes Data are mean ± SD; mean (SE); median (IQR); or (95% CI)		
					Baseline	Post-intervention	Between group differences
Randomised Controlled Trials:							
Henke, 2014, Germany [33]	• Centre-based aerobic and resistance • Chest physiotherapy	Assessments occurred after third chemotherapy cycle	Aerobic training five days per week. Resistance training on alternate days	nr	6MWT (metres):		
					IG: 378.4 ± 106.7 CG: 240.8 ± 150.5	IG: 397.1 ± 102.6 CG: 193.3 ± 162.8	*p* < 0.05
Mendizabal-Gallastegui, 2023, Spain [38]	• Centre-based aerobic and resistance	8	Three times per week	Percentage attended centre-based sessions: 14.3% attended more than 80% 57.1% attended more than half of sessions	6MWT (metres):		
					IG: 480.8 ± 86.4 CG: 493.8 ± 82.2	IG: 520.3 (503.6 to 536.9) CG: 504.5 (486.4 to 522.6)	24.8 (5.1 to 44.6)
					EORTC-QLQ-C30 physical functioning:		
					IG: 89.8 ± 12.2 CG: 92.2 ± 10.0	IG: 88.2 (83.2 to 93.2) CG: 91.2 (85.7 to 96.6)	7.7 (-0.01 to 15.4)
					SF-36 physical functioning:		
					IG: 79.2 ± 20.1 CG: 74.8 ± 23.8	IG: 42.4 (39.6 to 45.3) CG: 44.6 (41.6 to 47.5)	-1.8 (-5.2 to 1.4)
Mikkelsen, 2022, Denmark [39]	• Centre-based resistance • Home-based walking • Protein supplements • Individualised nurse-led counselling	12	Twice per week	Median adherence (IQR): Centre-based: 69% (21%-88%) Home-based: 75% (33%-100%)	6MWT (metres):		
					IG: 463.7 ± 98.1 CG: 434.4 ± 92.9	IG: 503.5 ± 91.4 CG: 438.5 ± 117.2	*p* = 0.002
					EORTC-QLQ-C30 physical functioning:		
					IG: 75.9 ± 19.6 CG: 78.1 ± 171	IG: 85.5 ± 12.4 CG: 81 ± 14.8	*p* = 0.55
Yee, 2019, Australia [55]	• Home-based aerobic and resistance	8 Follow-up: 16	Twice per week. Walking programme on non-training days	Centre-based rate: 100% Home-based rate: 25%	6MWT (metres):		
					IG: 531.4 ± 136.2 CG: 506.3 ± 93.9	IG: 40 ± 23 CG: -46 ± 56	86 (38 to 134) Effect size: 1.54 Glass’s delta > 0.8
					IPAQ (MET-min/week):		
					IG: 1709 ± 1785 CG: 1898 ± 2471	IG: 228 ± 915 CG: -738 ± 1622	966 (-514 to 2447) Effect size: 0.60 Glass’s delta < 0.8
					EORTC-QLQ-C30 physical functioning:		
					nr	IG: 5.8 ± 6.6 CG: -6.7 ± 7.3	12.5 (4.4 to 20.6) Effect size: 1.71 Glass’s delta > 0.8
Dhillon, 2017, Australia [29]	• Centre-based aerobic • Behaviour change workshop • Educational materials on exercise and nutrition	8 Follow-up: 16 and 24	Once per week	69% completed all physical activity sessions	6MWT (metres):		
					IG: 251.0 CG: 234.9	IG: 517.7 CG: 516.3 (-75.9 to 78.64)	*p* = 0.972
					Accelerometer (min/day):		
					IG: 13.18 CG: 15.62	IG: 18.05 CG: 13.24 (-4.12 to 13.73)	*p* = 0.289
					EORTC-QLQ-C30 physical functioning:		
					IG: 75.85 CG: 77.38	IG: 78.31 CG: 77.30 (-7.31 to 9.32)	*p* = 0.812
Edbrooke, 2019, Australia [30]	• Home-based aerobic and resistance	8 Follow-up: 24	Aerobic training twice per week Resistance training three times per week	Adherence rate: 65% completed 26/40 aerobic sessions 53% completed 21/40 resistance sessions	6MWT metres:		
					IG: 467.0 ± 117.6 CG: 482.7 ± 115.9	nr	-25.36 (-63.98 to 13,26) Effect size: 0.29 *p* = 0.198
					IPAQ (MET-min/week):		
					IG: 294.5 (99.0–94.0) CG: 235.5 (0.0–834.0)	nr	-317.59 (-1314.09 to 678.91) Effect size: 0.14 *p* = 0.838
					Accelerometer (steps):		
					IG: 2859.6 (2034.0–3849.2) CG: 3195.2 (2161.3–4839.0)	nr	174.49 (-1504.66 to 1853.65) Effect size: 0.05 *p* = 0.838
Rutkowska, 2019, Poland [46]	• Centre-based aerobic and resistance • Chest physiotherapy • Relaxation training	4	Five days per week for two weeks	Inpatient programme adherence: 100%	6MWT (metres):		
					IG: 486 ± 92 CG: 487 ± 100	IG: 531 ± 103CG: 490 ± 124	*p* = 0.09
Scott, 2018, USA [49]	• Centre-based aerobic	12	Three times per week	Mean adherence ± SD: 63% ± 30 (range 0%-100%)	6MWT (metres):		
					IG: 504 ± 96 CG: 501 ± 98	IG: 533 ± 90 CG: 530 ± 103	*p* = 0.89
Uster, 2018, Switzerland [52]	• Centre-based aerobic and resistance • Nutritional intervention	12 Follow-up: 24	Twice per week	Mean adherence ± SD: 16 ± 7 (67%)	6MWT (metres):		
					nr	nr	*p* > 0.05
					EORTC-QLQ-C30 physical functioning:		
					nr	IG: 0 ± 3.3 CG:—8.7 ± 3.8	*p* = 0.34
Zimmer, 2018, Germany [57]	• Centre-based aerobic, resistance and balance	8 Follow-up: 12	Twice per week	Mean training frequency: 88.3%	6MWT (metres):		
					IG: 477.7 ± 91.9 CG: 459.7 ± 74.1	IG: 502.2 ± 62.1 CG: 478.2 ± 75.2	*p* = 1.00
Cormie, 2013, Australia [26]	• Centre-based resistance • Home-based aerobic	12	Twice per week	70% of participants completed 24/24 sessions. 83% completed 20/24 sessions	6 m-WT (seconds):		
					IG: 4.48 ± 0.54 CG: 4.45 ± 0.56	IG: 4.23 ± 0.33 CG: 4.76 ± 0.42	-0.55 (-0.78 to -0.32) *p* < 0.001
					400MWT (seconds):		
					IG: 252.1 ± 40.8 CG: 280.8 ± 53.0	IG: 246.9 ± 32.9 CG: 286.5 ± 50.5	-13.7 (-23.5 to -3.9) *p* = 0.010
					Accelerometery (min/ week):		
					IG: 341.7 ± 143.3 CG: 359.6 ± 140.7	IG: 356.7 ± 112.6 CG: 316.8 ± 121.4	82.5 (31.8 to 133.2) *p* = 0.003
					TUG (seconds):		
					IG: 7.41 ± 1.50 CG: 7.59 ± 1.91	IG: 6.97 ± 1.02 CG: 7.32 ± 1.17	-0.42 (-1.00 to 0.12) *p* = 0.150
					SF-36 physical functioning (NBS) ± SD:		
					IG: 44.2 ± 9.0 CG: 45.0 ± 11.4	IG: 46.5 ± 9.4 CG: 45.8 ± 7.8	0.0 (-4.2 to 4.2) *p* = 0.996
Galvão, 2018, Australia [31]	• Centre-based aerobic and resistance	12	Three times per week	Participants completed a mean ± SD: 32 ± 10 out of 36 exercise sessions	6 m-WT (seconds):		
					IG: 4.5 ± 0.9 CG:4.6 ± 1.1	IG: 4.8 ± 1.0 CG: 4.6 ± 1.3	0.2 (-0.1 to 0.4) *p* = 0.192
					TUG (seconds):		
					IG: 7.5 ± 2.4 CG: 6.9 ± 1.6	IG: 7.5 ± 2.5 CG: 6.8 ± 1.4	0.1 (-0.3 to 0.6) *p* = 0.497
					400MWT (seconds):		
					IG: 249.1 ± 38.7 CG: 252.0 ± 47.7	IG: 245.2 ± 32.9 CG: 249.3 ± 41.0	-1.6 (-8.7 to 5.5) *p* = 0.641
					SF-36 physical functioning:		
					IG: 47.8 ± 6.8 CG: 45.5 ± 8.2	IG: 49.5 ± 5.0 CG: 44.8 ± 7.8	3.2 (0.4 to 6.0) *p* = 0.028
Maddocks, 2009, UK [36]	• Centre/ home-based electrotherapy	4	Daily NMES encouraged	Participants usage of NMES median (range): 80% (69%-100%) of overall recommended treatment time	ESWT (metres):		
					IG: 660 ± 550 CG: 845 ± 517	IG: -20 ± 254 CG: -159 ± 222	138 (-118 to 394) *p* = 0.27
					ActivPAL (steps):		
					IG: 5061 ± 1516 CG: 5554 ± 4581	IG: 136 ± 2660 CG: -633 ± 1335	768 (-1530 to 3066) *p* = 0.48
Oldervoll, 2011, Norway [41]	• Centre-based aerobic and resistance	8	Twice per week	Adherence rate to centre-based programme: 69%	SWT (metres):		
					IG: 339 ± 17.1 CG: 390 ± 17.8	IG: 380 ± 24.2 CG: 369 ± 21.5	60 (16.0 to 103.4) *p* = 0.008
Stuecher, 2019, Germany [50]	• Home-based walking	12	Three-five times per week	Mean exercise adherence rate: 81.3%	SPPB (points):		
					IG: 9.4 ± 2.3 CG: 8.1 ± 2.6	IG: 0.42 ± 1.16 CG: 0.08 ± 2.72	*p* = 0.36
Maddocks, 2013, UK [37]	• Home-based electrotherapy	8–11 depending on chemo-therapy cycle	Daily NMES encouraged	50% of participants met the minimum adherence criterion	Accelerometery (steps):		
					IG: 3163 (2267–3855) CG: 3362 (2818–4644)	IG: 3362 (2818–4644) CG: 3332 (2636–4429)	51 (-1736 to 238) *p* = 1.00
					EORTC-QLQ-C30 physical functioning:		
					IG: 73 (40–87) CG: 87 (73–93)	IG: 67 (67–80) CG: 80 (60–87)	0 (-13 to 13) *p* = 0.47
Bade, 2021, USA [23]	• Home-based walking • Education session	12	Daily walking	nr	Pedometer (steps range):		
					4707 (1568–12,222)	Week 6: 5605 (1079–9764)	*p* = 0.87
						Week 12: 4606 (746–10,238)	
					EORTC-QLQ-C30 physical functioning (SE):		
					IG: 83.0 (17) CG: 82.8 (14.5)	IG: 88.1 (3.1) CG: 88.8 (3.3)	*p* = 0.85
Cheville, 2013, USA [25]	• Home based aerobic and resistance	8	Four REST sessions per week	76.9% completed recommended levels of exercise programme	AM-PAC Mobility (points):		
					nr	IG: 4.88 ± 4.66 (2.96 to 6.80) CG: 0.23 ± 5.22 (-1.76 to 2.22)	*p* = 0.002
Cheville, 2019^b^, USA [17]	• Home-based aerobic and resistance • Automated monitoring of symptoms either online/ telephone • Outpatient physiotherapy referral	24	Four REST sessions per week	nr	AM-PAC Mobility (points):		
					IG: 60.2 ± 3.7 CG: 60.7 ± 3.5	nr	1.3 (0.08 to 2.35) *p* = 0.03
					EQ-5D-5L (points):		
					IG: 0.8 ± 0.1 CG: 0.8 ± 0.1	nr	0.04 (0.004 to 0.071) *p* = 0.01
Non-Randomised Controlled Trials:							
Schink, 2018, Germany [47]	• Centre-based WB-EMS • Nutritional input from dietician	12	Twice per week	Adherence rate with programme: 86.6% ± 10.9	6MWT (metres):		
					IG: 521.6 ± 104.5 CG: 484.30 ± 135.0	IG: 577.1 ± 95.4 CG: 504.6 ± 116.8	*p* = 0.036
					EORTC-QLQ-C30 physical functioning:		
					IG: 78.55 ± 20.12 CG: 74.37 ± 20.81	IG: 80.47 ± 20.90 CG: 77.32 ± 15.51	*p* = 0.542
Schink, 2020, Germany [48]	• Centre-based WB-EMS • Nutritional input from dietician	12	Twice per week	nr	6MWT (metres):		
					IG: 543.8 ± 99.5 CG: 550.1 ± 85.5	nr	44.57 (13.83 to 75.30) *p* = 0.006
					EORTC-QLQ-C30 physical functioning:		
					IG: 76.3 ± 24.2 CG:78.7 ± 16.64	nr	9.30 (-0.69 to 19.30) *p* = 0.67
Zhao, 2016, USA [56]	• Centre and home based aerobic and resistance	14	Up to three times per week. Encouraged to complete home programme a minimum of five days per week	Centre-based adherence rate: 72%	6MWT (feet):		
					IG: 1400 ± 243 CG: 1530 ± 233	IG: 60 (40) CG: -19 (89)	*p* > 0.05
					TUG (seconds):		
					IG: 8 ± 3 CG: 8 ± 1	IG: -0.7 (0.6) CG: -0.2 (0.6)	*p* > 0.05
					PASE:		
					IG: 147 ± 90 CG: 150 ± 116	IG: 42 (18) CG: -10 (31)	*p* > 0.05
					SF-36 physical component:		
					IG: 67 ± 20 CG: 56 ± 23	IG: 1 (4) CG: -3 (8)	*p* > 0.05
Randomised Comparative:							
Litterini, 2013, USA [35]	• Centre-based aerobic versus resistance	10	Twice per week	70% of participants attended 14/20 sessions	SPPB (points):		
					CVG: 9.77 ± 2.25 RG: 9.38 ± 2.10	CVG: 10.45 ± 2.05 RG: 9.91 ± 1.95	0.75 (0.44 to 1.06) *p* < 0.001
Randomised Cross-Over:							
Vanderbyl, 2017, USA [54]	• Centre-based Qigong • Centre-based aerobic and resistance	Total: 14 Each intervention: 6 with 2 weeks break between interventions	Twice per week	Mean adherence ± SD: Qigong:75.1% ± 21.9 SET: 86.8% ± 12.5	6MWT (metres):		
					QG: 430.6 ± 66.2 SET: 420.0 ± 85.8	QG: -4.0 ± 45.7 SET: 73.3 ± 60.1	*p* = 0.002 (favouring SET)
Single Arm Studies:							
Avancini, 2023, Italy [21]	• Centre-based aerobic and resistance	12	Twice per week	Overall adherence: 84% Aerobic component: 85% Resistance component: 82%	6MWT (metres):		
					528.3 ± 82.1	564.8 ± 69.8	*p* = 0.021
					EORTC-QLQ-C30 physical functioning:		
					84.4 ± 13.4	89.44 ± 9.6	*p* = 0.108
Avancini, 2024, Italy [22]	• Centre/ home-based aerobic and resistance	12	Twice per weekly	Overall adherence: 88%	6MWT (metres):		
					489.0 ± 79.5	519.1 ± 71.4	34.51 (19.82 to 49.21) *p* < 0.001
					EORTC-QLQ-C30 physical functioning:		
					80 (73.3–93.3)	86.7 (80.0–93.3)	*p* = 0.002
Chasen, 2013, USA [24]	• Centre-based aerobic and resistance • MDT assessment	8	Twice per week	nr	6MWT (metres):		
					367.4 ± 123.2	422.7 ± 127.6	Cohen’s d: 0.80* p* < 0.001
					TUG (seconds):		
					11.4 ± 6.5	9.1 ± 3.9	Cohen’s d: 0.65* p* < 0.001
Delrieu, 2020, France [28]	• Home-based walking	24	Daily walking	77% (95% CI: 62.2 to 88.5) achieved the recommended levels of physical activity	6MWT (metres):		
					451.6 ± 99.7	482.6 ± 106.3	*p* < 0.001
					IPAQ (MET-mins/week):		
					795.6 ± 1073.6	944.3 ± 1013.9	*p* = 0.17
					EORTC-QLQ-C30 physical functioning:		
					76.3 ± 22.4	82.0 ± 17.1	*p* = 0.17
Kuehr, 2014, Germany [34]	• Centre/ home-based aerobic and resistance	8 Follow-up: 16	Inpatient: Five times per week Community: Three times per week	Overall adherence rate to exercise programme: 82% Inpatient adherence: 95% Home programme adherence: 77%	6MWT (metres):		
					493 ± 100	525 ± 95	*p* < 0.01
O’Connor, 2020, Ireland [40]	• Home-based electrotherapy	4	Incrementally increasing from two sessions (week one) to five sessions (week four). Total of 14 sessions over four weeks	Mean number of completed NMES sessions: 12 ± 3	6MWT (metres):		
					232 ± 69	309 ± 61	*p* = 0.040
					TUG (seconds):		
					37.6 ± 17.1	14.9 ± 6.1	*p* = 0.399
					EORTC-QLQ-C30 physical functioning:		
					70 (38–90)	63 (55–72)	*p* = 0.725
Ozalevli, 2010, Turkey [42]	• Centre-based resistance • Chest physiotherapy • TENS	Number of sessions: 24.61 ± 15.71	Daily for length of inpatient stay	Adherence rate with programme: 100%	6MWT (metres):		
					246.39 ± 162.75	321.39 ± 178.70	*p* = 0.003
					NHP physical mobility:		
					35.27 ± 27.78	22.74 ± 23.33	*p* = 0.03
Park, 2019, South Korea [43]	• Home-based aerobic and resistance	12	Daily exercise encouraged	Mean exercise sessions per week ± SD: Week 1: 3.8 ± 1.2 Week 6: 4.2 ± 1.1 Week 12: 4.1 ± 1.2	6MWT (metres):		
					384.2 ± 74.6	447.4 ± 50.4	*p* < 0.001
					EORTC-QLQ-C30 physical functioning:		
					78.2 ± 14.3	81.1 ± 15.7	*p* = 0.06
Quist, 2012, Denmark [44]	• Centre-based aerobic and resistance • Home-based walking	6	Twice per week. Home programme encouraged three times per week	Mean participation: Centre-based intervention: 73.3% (range: 45%-100%) Home programme: 8.7%	6MWT (metres):		
					524.7 ± 88.5	564.0 ± 88.6	39.3 (12.5 to 66.1) *p* = 0.006
Quist, 2015, Denmark [45]	• Centre-based aerobic and resistance	6	Twice per week	Mean participation in the intervention: 68% (range: 45%-100%)	6MWT (metres):		
					527.4 ± 121.5	561 ± 124.7	(20.3 to 47.0) *p* < 0.0001
Van Den Dungen, 2014, The Netherlands [53]	• Centre-based aerobic and resistance	6	Three times per week	85% of participants completed 8/12 centre-based sessions	6MWT (metres):		
					435.0 ± 135.2	480.0 ± 137.0	*p* < 0.01
					EORTC-QLQ-C30 physical functioning:		
					76.9 ± 25.1	78.5 ± 23.3	*p* = 0.33
Temel, 2009, USA [51]	• Centre-based aerobic and resistance	8–12	Twice per week	Completion rate: 44%	6MWT (metres):		
					410.55 ± 83.28	435.73 ± 72.66	-57.25 to 6.89 *p* > 0.05
Cormie, 2014, Australia [27]	• Centre-based aerobic and resistance	12 Follow-Up: 24	Twice per week	Participants attended 20.4 ± 6.9 out of 24 sessions	6 m-WT (seconds):		
					4.59 ± 0.45	4.32 ± 0.37	-0.27 (-0.39 to -0.15) *p* < 0.001
					TUG (seconds):		
					7.2 ± 1.3	6.9 ± 1.3	-0.26 (-0.62 to 0.10) *p* = 0.147
					400MWT (seconds):		
					262.6 ± 43.6	255.4 ± 43.4	-7.2 (-12.0 to -2.3) *p* = 0.007
					SF-36 physical functioning:		
					44.2 ± 9.4	46.2 ± 7.8	2.0 (-0.4 to 4.3) *p* = 0.095
Hanson, 2023, USA [32]	• Home-based aerobic and resistance	12	Two-four times per week	Self-reported adherence: Walking programme: 79.8% Resistance programme: 63.4%	6 m-WT (seconds):		
					4.4 ± 1.5	4.6 ± 1.5	0.1 (-0.2 to 0.4) *p* = 0.378
					TUG (seconds):		
					10.5 ± 9.2	10.9 ± 11.1	0.4 (-1.0 to 1.7) *p* = 0.629
					400MWT (seconds):		
					329.8 ± 97.8	313.5 ± 87.3	-16.3 (-36.9 to 4.3) *p* = 0.111
					SPPB (points):		
					10.4 ± 2.2	11.0 ± 1.8	0.6 (-0.2 to 1.3) *p* = 0.157

*nr* = not reported

Cohen’s d: Small effect = 0.2. Medium effect = 0.5. Large effect = 0.8

Glass’s delta > 0.8 = large effect size

Mean scores and CIs presented were tabulated as reported in the referenced studies. The studies have been organised based on their study design, respective outcome measures and further ordered according to whether the intervention favoured the intervention group

*400MWT* 400 Metre Walk Test, *6 m-WT* 6 Metre Walk Test, *6MWT* 6 Minute Walk Test, *AM-PAC* Ambulatory Post Acute Care, *CG* Control group, *CI* Confidence interval, *CVG* Cardiovascular group, *EORTC QLQ C-30* European Organisation for Research and Treatment of Cancer Quality-of-Life Questionnaire Core 30, *ESWT* Endurance Shuttle Walk Test, *IG* Intervention group, *IPAQ* International Physical Activity Questionnaire, *IQR* Interquartile range, *MET* Maximal metabolic equivalent, *MDT* Multidisciplinary team, *NHP* Nottingham Health Profile, *NMES* Neuromuscular electrical stimulation, *PASE* The Physical Activity Scale for the Elderly, *QG* Qigong group, *REST* Rapid, Easy, Strength Training, *RG* Resistance group, *SD* Standard deviation, *SE* Standard error, *SET* Standard exercise training, *SF-36* Short-Form 36, *SPPB* Short Physical Performance Battery, *SWT* Shuttle Walk Test, *TENS *Transcutaneous electrical nerve stimulation, *TUG* Timed Up and Go, *WB-EMS* Whole-body electromyostimulation

### Non-Pharmacological interventions

#### Exercise

Thirty three studies included an exercise component [17, 21–35, 38, 39, 41–46, 49–57]. In 25 of these studies, exercise was assessed as a standalone intervention [17, 21–23, 25–28, 30–32, 34, 35, 38, 41, 43–45, 49, 51, 53–57]. In eight studies exercise was evaluated in conjunction with one or more complementary non-pharmacological approaches including: nutritional support [24, 29, 39, 52], psychosocial support [24, 39], education sessions/ materials [23, 29], electrotherapy [42], and manual techniques delivered by therapists [33, 42, 46].

### Resistance exercise

One study [35] investigated a resistance training programme as a standalone intervention. They conducted a 10-week randomised comparative study evaluating an aerobic programme versus a resistance training programme. They found that both resistance (baseline: 9.38 ± 2.10 points; post-intervention 9.91 ± 1.95 points) and cardiovascular (baseline: 9.77 ± 2.25 points; post-intervention: 10.45 ± 2.05 points) training resulted in statistically significant improvements in the Short Physical Performance Battery, without substantial differentiation between the exercise types [35].

### Aerobic exercise

Six studies evaluated an aerobic based intervention [23, 28, 29, 35, 49, 50], with two studies finding a significant change in mobility following aerobic training [28, 35]. Three studies evaluated walking programmes [23, 28, 50], one evaluated treadmill training [49], one evaluated an aerobic programme alongside nutritional and behaviour change advice [29], and one evaluated an aerobic programme versus a resistance programme [35]. The intensity and frequency of training varied between studies. For example, in two walking programme studies [23, 28] specific step-count goals were utilised, with one study aiming for a weekly increase of 400 daily steps over 12 weeks [23], whilst the other aimed for a weekly increase of 1000 daily steps over six-months [28]. Participants who already achieved ≥ 10,000 steps per day were encouraged to maintain their activity levels. The programme with the longer duration and higher step-count goal demonstrated statistically significant improvements in 6MWT (baseline 451.6 ± 99.7; post-intervention 482.6 ± 106.3; *p* < 0.001) [28]. The other walking programme required participants to walk for 150 min per week over 12 weeks, but found no significant improvement in mobility [50]. Another study conducted a twice-weekly centre-based intervention and found a positive impact on clinical measures of mobility as described earlier [35]. An eight-week multicomponent aerobic based programme and a treadmill based intervention over 12 weeks found no statistically significant improvement in mobility outcomes [29, 49].

### Combined aerobic and resistance exercise

Twenty seven studies assessed exercise programmes that combined both aerobic and resistance components [17, 21, 22, 24–27, 30–34, 38, 39, 41–46, 51–57], with 19 of these reporting improvements in mobility outcome(s) [17, 21, 22, 24–27, 33, 34, 38, 39, 41–45, 53–55]. Two studies included three mobility outcomes, with improvements seen in two of the tools [26, 27]. Programmes typically targeted major muscle groups in the trunk, upper limbs, and lower limbs, though repetitions, exercise intensity and recommended activity levels differed. Five studies integrated exercise with other interventions, including nutritional interventions [39, 52], counselling [39], referrals to physiotherapy [17], electrotherapy [42] and breathing exercises combined with manual chest physiotherapy techniques [42, 46].

Six of these studies were home-based [17, 25, 30, 32, 43, 55]. Two of these studies reported a significant improvement in 6MWT in the intervention group following a 12 week intervention in one study (baseline: 384.2 ± 74.6 m; post-intervention: 447.4 ± 50.4 m; *p* < 0.001) [43] and an eight-week intervention in the other (baseline: 531.4 ± 136.2 m; post-intervention mean change: 40 ± 23 m) [55]. Two studies reported statistically significant improvements in AM-PAC mobility scores in the intervention groups [17, 25]. One of these studies, evaluated a home-based combined exercise programme over eight-weeks (mean difference 4.88 ± 4.66 points; *p* = 0.002) [25], whilst the other evaluated a six-month telerehabilitation intervention comprising of a combined home-based exercise programme and outpatient physiotherapy referral (baseline 60.2 ± 3.7 points; post-intervention between group difference 1.3 points; *p* = 0.03) [17].

Thirteen studies were conducted in a centre-based setting [21, 24, 31, 33, 38, 41, 45, 46, 51, 52, 52, 53, 57], with seven reporting significant changes in mobility outcomes [24, 33, 38, 41, 42, 45, 53]. Eight studies [22, 26, 27, 34, 39, 44, 54, 56] evaluated a combination of home and centre-based interventions with studies showing positive changes in some [26, 27] or all of the mobility outcomes [22, 34, 39, 44, 54]. Participants received more frequent contact with the study team in centre-based interventions (twice to five times per week) than home-based interventions (twice per week to bi-monthly). Only six studies included follow-ups [27, 30, 34, 52, 55, 57]. This ranged from four weeks to six months, with continued improvements found in two studies, eight weeks [55] and six months [27] post-intervention.

### Electrotherapy

Five studies evaluated the efficacy of electrotherapy [36, 37, 40, 47, 48], with three studies finding significant improvements in mobility outcomes [40, 47, 48]. Electrotherapy protocols varied greatly in terms of stimulation site, frequency (Hz), session number and overall duration. In two studies, dietary advice was combined with whole-body electrical muscle stimulation (WB-EMS) during active range of motion activities and applied to major muscle groups [47, 48]. The remaining three studies evaluated neuromuscular electrical stimulation (NMES) as a single component intervention [36, 37, 40]. One study utilised transcutaneous electrical nerve stimulation in conjunction with exercise [42], but used this modality for pain relief rather than functional gains and was consequently categorised as a multi-component exercise intervention rather than an electrotherapy-based intervention.

Two studies [36, 37] encouraged daily NMES usage within their studies, targeting the quadriceps but found no improvements in mobility outcomes. One study [40] recommended a progressive increase use of NMES over the four week study targeting the quadriceps and hamstrings with a combination of low and high frequency stimulation. The study found statistically significant improvements in 6MWT (baseline: 232 ± 69 m; post-intervention: 309 ± 61 m; *p* = 0.040) but no statistically significant improvement in TUG [40]. Two studies [47, 48] recommended at least two days rest between WB-EMS training to allow for muscle recovery and opted for twice weekly training sessions, with participants wearing a vest, hip belt, upper arm, and thigh cuffs with integrated electrodes. Both studies found statistically significant improvements in the interventions group’s 6MWT scores (baseline: 521.6 ± 104.5 m; post-intervention 577.1 ± 95.4 m; *p* = 0.036 [47]; baseline 543.8 ± 99.5 m; post-intervention coefficient 44.57 m; 95% CI 13.83 to 75.30; *p* = 0.006 [48]).

### Resources

There was wide variation in staffing levels, settings, equipment, and essential resources required to deliver the intervention. Physiotherapists delivered the intervention in 13 studies [17, 25, 33, 36, 41, 42, 44–46, 51–54], seven were led by exercise specialists [28, 29, 34, 50, 55–57], five studies were participant-led [23, 37, 40, 43, 50], four were led by physiologists [26, 27, 31, 49], three studies involved a multidisciplinary team [24, 47, 48], two studies were overseen by a kinesiologist [21, 22] and one study was nurse led [38]. Studies varied greatly in intervention frequency, with some studies recommending daily completion of the programme [23, 28, 36, 37, 42, 43] whilst others opted for weekly [29]. Typically, studies that relied on clinicians to deliver the intervention opted for a frequency of two to three sessions per week. However, one study required a high staffing commitment, with participants receiving inpatient physiotherapy five days per week over four weeks [46], whilst another delivered the intervention twice per day for the duration of the participant’s inpatient stay [42].

Studies were conducted in various settings, with 26 studies requiring participants to attend a hospital, clinic or community centre to undertake the intervention [21, 24, 26, 27, 29, 31, 33–36, 38, 39, 41, 42, 44–49, 51–54, 56, 57], whilst 12 studies delivered a home-based intervention [17, 22, 23, 25, 28, 30, 32, 37, 40, 43, 50, 55]. Regarding equipment, 15 studies used high-tech aerobic equipment such as rowers, cycle ergometers and treadmills [21, 22, 31, 34, 35, 39, 41, 44–46, 49, 51–53, 57], nine studies used resistance bands [21, 22, 32–34, 38, 41, 43, 55], five studies used free weights [34, 35, 38, 55, 56], nine studies provided activity monitors [23, 25, 28, 29, 32, 39, 43, 50, 55], and six studies used electrotherapy devices [36, 37, 40, 42, 47, 48].

### Contextual factors

Geographical contextual factors were identified as potential barriers to participation in 18 studies [24–27, 31, 33, 35, 36, 38, 39, 41, 44, 45, 47, 48, 51, 52, 56]. Participants reportedly faced transportation and parking challenges when traveling to healthcare facilities for the intervention, as highlighted in one study [24]. Two studies determined participants' eligibility based on the participant’s reported ability to attend the intervention sessions twice weekly, leading to those living too far away from the study centre to be allocated to the control group or excluded from the study [47, 48].

In terms of socioeconomic, sociocultural and epidemiological factors, most studies were conducted in affluent Western countries. In studies that reported ethnicity, ≥ 80% of the study population were white [17, 22, 23, 25, 32, 35, 51], with native language proficiency forming part of the inclusion criteria in 12 studies [17, 25, 28–30, 34, 35, 39, 50, 51, 53, 55]. One study had specific technological requirements i.e. the participant was required to own a specific smartphone and be able to effectively utilise their app [43].

## Discussion

### Main findings

This review aimed to provide a comprehensive synthesis of non-pharmacological interventions that evaluated mobility in people with advanced cancer. The review included 38 randomised and non-randomised studies with 2464 participants overall. Our main findings were: i) both exercise and neuromuscular electrical stimulation interventions had an overall positive impact on mobility outcomes; ii) we identified a disparity between clinical and patient-reported measures in detecting changes in mobility status. Observed improvements in clinical measurement tools assessing mobility status were not always reflected in patient-reported outcomes when measured in parallel; iii) regarding resources and context, the centre-based nature of many interventions as well as a requirement for native language proficiency, may have limited access to, and inclusivity of, interventions for this group.

### Interventions

Our findings suggest exercise and neuromuscular electrical stimulation interventions may help optimise mobility among people with advanced cancer. However, the heterogeneity across studies precluded meta-analysis, so the narrative synthesis findings should be interpreted with due caution.

Exercise-based studies typically focused on the physical domain of mobility, targeting areas such as muscle strength, endurance, and flexibility. Theoretically, interventions targeting symptoms such as breathlessness, fatigue, pain, nutrition and psychosocial domains may indirectly impact on mobility [59, 60]. For example, holistic breathlessness services aim to reduce breathlessness, which may positively influence the psychosocial mechanisms described within Webber and colleagues’ model of mobility [6], such as confidence and self-efficacy [61]. Additionally, occupational therapy interventions, such as home modifications and provision of assistive devices align with the psychosocial and environmental domains of Webber and colleagues’ model [6], and may influence factors such as falls risk, promote energy conservation, and influence an individual's capacity and willingness to mobilise [62]. Notably, these types of single component interventions were excluded as many did not use mobility measures [63–65] or sub-analysis of mobility outcomes were not reported within study results [66]. Future studies that directly or indirectly target mobility, should incorporate outcome measures that capture changes across the multiple domains of mobility.

### Measurement

Studies in our review employed a combination of clinical tools and PROM. Clinical measures, such as the 6MWT, evaluate the impact of interventions on exercise capacity and serve as good predictors of community mobility [67], but solely measure the physical domain of mobility. PROM such as the EORTC-QLQ-C30 and SF-36 focus on health-related quality of life, but both assess different domains of mobility. We had anticipated that improvements in clinical measures, like the 6MWT, would equate to enhanced physical function in the PROM [68]. However, our review reveals that improvements in the clinical measures of mobility were not always reflected in PROM. This discrepancy may be attributed to limited statistical analysis and reliance on vote counting. Alternatively, the discrepancy may be linked to most studies evaluating interventions targeting the physical domain of mobility, whereas the PROM, even though assessing physical function, include various interconnected mobility domains [69], such as psychosocial and environmental factors [6]. As a result, improvements in the specific physical clinical measures might not be reflected in the broader aspects of mobility assessed within the PROM.

Moreover, PROM such as the EORTC-QLQ-C30 and SF-36 may not capture the nuances of mobility in a natural setting [70]. These instruments focus on assessing mobility domains situated within the "Activities and Participation" component of the International Classification of Functioning, Disability, and Health (ICF) framework [70, 71]. However, within this ICF component, these PROMs offer limited evaluation of mobility concerning community and social participation, domestic life, and the ability to mobilise in different settings [70]. Psychological, emotional and social factors contribute to an individual’s walking experience [72], but current measurement approaches, which particularly rely on clinical measures, may not fully capture the diverse dimensions of mobility. Only a few studies in our review evaluated mobility in natural settings, where individuals navigate domestic life, engage in community activities and experience the broader facets of mobility. Tools such as the PROMIS Cancer Item Bank for Physical Function, AM-PAC, World Health Organisation Disability Assessment Schedule (WHODAS), and PASE, may offer a more comprehensive assessment of mobility in people with advanced cancer [70].

### Access and inclusivity

The geographical considerations highlighted in this review emphasise challenges associated with centre-based interventions, including distance from the site, transportation, and parking. A majority of studies were conducted in large metropolitan areas, potentially limiting the generalisability of findings to rural or remote populations [73]. Exploring alternative delivery methods, particularly for those benefiting from non-pharmacological interventions but facing access challenges, is crucial. The effectiveness of tele-rehabilitation, catalysed further by the recent COVID-19 pandemic, underscores the potential for alternative healthcare modalities [17]. A third of the reviewed studies investigated home or community-based interventions, incorporating telephone and/or online support, with 45% showing significant improvements in mobility. Whilst telerehabilitation in advanced cancer has shown to be cost-effective [74], further research is needed to compare outcomes across various delivery models and assess their impact on factors such as quality of life [75].

Regarding inclusivity, the seven studies that reported ethnicity revealed a significant overrepresentation of white participants (≥ 80%). While this may be representative of the local population, programmes should actively eliminate barriers to inclusivity, ensuring equitable representation for traditionally underserved and underrepresented populations in both research and healthcare [76].

### Considerations for future research

While exercise and electrotherapy interventions suggest positive impacts on mobility, there is a significant gap in addressing the broader concept of mobility beyond physical functioning. Future studies should integrate the various domains in Webber and colleagues’ model [6], acknowledging their interconnected nature and influence on mobility, whilst also considering geographical, sociocultural and socioeconomic factors that may impact on access and inclusion. Integrating secondary measures like PROM that assess mobility within a natural setting, will offer a comprehensive understanding of these interconnected domains. Moreover, the absence of single component interventions, such as holistic breathlessness services and occupational therapy, underscores the need to explore these areas to understand their potential impact on mobility.

### Strengths and limitations

This review adheres to the recommendations outlined in the PRISMA statement [15]. Transparency in reporting was upheld through the development of a comprehensive study protocol, and to minimise judgment errors and bias, screening and data extraction were conducted independently by two or more authors. Some limitations also warrant consideration. Firstly, due to the level of heterogeneity of the included studies, a metanalysis was not suitable. The selected method of vote counting, grounded in statistical significance, offers limited insights into the magnitude of effects and does not consider variations in the relative sizes of individual studies [77]. Additionally, studies characterised by inadequate statistical power, which do not sufficiently exclude clinically significant effects, risk being counted as not demonstrating a therapeutic benefit [77]. Secondly, the inclusion criteria, requiring studies to have ≥ 95% of their sample composed of individuals with advanced cancer, led to the exclusion of studies that nearly met this threshold, and may have resulted in the omission of valuable data. Lastly, due to a lack of resources, a risk of selection bias exists, as only studies published in English were included.

## Conclusion

This systematic review suggests a positive impact of both exercise and neuromuscular electrical stimulation interventions on mobility outcomes. However, included studies were mostly conducted in high resource countries and may not be generalisable to other settings. Opportunities for future research include the use of mobility outcomes to evaluate the impact of tailored interventions targeting different domains of mobility. Population and contextual factors should be carefully considered to promote inclusivity and to eliminate barriers for diverse populations.

## Supplementary Information

Below is the link to the electronic supplementary material.

Supplementary file1 (DOCX 44 KB)

## Data Availability

No datasets were generated or analysed during the current study.
