# Supplementary material for: Non-pharmacological interventions targeting mobility among people with advanced cancer: a systematic review
Source: Support Care Cancer. 2024 Aug 5;32(9):569. doi: 10.1007/s00520-024-08767-x (PMC11300630; doi:10.1007/s00520-024-08767-x)
Supplement: Supplementary file 1 — Supplementary file1 (DOCX 44 KB) [file 520_2024_8767_MOESM1_ESM.docx]

**Non-pharmacological interventions targeting mobility among people with advanced cancer: a systematic review**

Carmine Petrasso^1*^, Joanne Bayly^1^, Simona Arculeo^2^, Megan Bowers^1^, Stefania Costi^3,4^, Lise Nottelmann^5,6^, Elena Turola^7^, Elisa Vanzulli^2^, Matthew Maddocks^1^

1 Cicely Saunders Institute of Palliative Care, Policy and Rehabilitation, King’s College London, London, England, UK

2 Fondazione IRCCS Istituto Nazionale dei Tumori, Milano, Italy

3 Physical Medicine and Rehabilitation Unit, Azienda USL – IRCCS di Reggio Emilia, Reggio Emilia, Italy

4 Surgical, Medical and Dental Department of Morphological Sciences, University of Modena and Reggio Emilia, Reggio Emilia, Italy

5 Research Unit for General Practice, Aarhus University, Aarhus, Denmark

6 The Research Unit, Department of Palliative Medicine, Bispebjerg Hospital, København, Denmark

7 Research and Statistics Infrastructure, Azienda USL – IRCCS di Reggio Emilia, Reggio Emilia, Italy

*corresponding author at: Carmine Petrasso, Cicely Saunders Institute of Palliative Care, Policy and Rehabilitation Bessemer Road, London, SE5 9PJ, UK. Email: [carmine.1.petrasso@kcl.ac.uk](mailto:carmine.1.petrasso@kcl.ac.uk)

**Supplementary Material**

Search strategies for electronic databases

Table S1: Search strategy for MEDLINE database:

| #1 | exp Neoplasms |
| --- | --- |
| #2 | neoplasm*.mp. |
| #3 | cancer*.mp. |
| #4 | advanced cancer*.mp. |
| #5 | metastat* cancer*.mp. |
| #6 | incurable cancer*.mp. |
| #7 | terminal cancer*.mp. |
| #8 | tumo?r*.mp. |
| #9 | palliative cancer*.mp. |
| #10 | end stage cancer*.mp. |
| #11 | Stage 3 cancer*.mp. |
| #12 | Stage 4 cancer*.mp. |
| #13 | 1 or 2 or 3 or 4 or 5 or 6 or 7 or 8 or 9 or 10 or 11 or 12 |
| #14 | exp Rehabilitation Centers/ or exp "Physical and Rehabilitation Medicine"/ or exp Rehabilitation Research/ or exp Rehabilitation/ or exp Hospitals, Rehabilitation |
| #15 | rehabilitation.mp. |
| #16 | exp Exercise/ or exp Exercise Therapy/ or exp Exercise Movement Techniques/ or exp Circuit-Based Exercise/ |
| #17 | exercis*.mp. |
| #18 | activit* adj2 (motor or physical).mp. |
| #19 | exp Resistance Training/ or exp “Physical Education and Training”/ |
| #20 | resistance train*.mp. |
| #21 | exp Patient Education as Topic/ |
| #22 | exp Health Education/ |
| #23 | exp Endurance Training/ |
| #24 | exp Activities of Daily Living/ |
| #25 | endurance train*.mp. |
| #26 | exp Physical Fitness |
| #27 | physical fitness.mp. |
| #28 | aerobic exercise*.mp. |
| #29 | exp Physical Therapy Modalities/ |
| #30 | physical therap*.mp. |
| #31 | physiotherap*.mp. |
| #32 | exp Occupational Therapy/ |
| #33 | occupational therap* |
| #34 | exp Electric Stimulation Therapy/ |
| #35 | electrotherapy.mp. |
| #36 | gait adj2 (training or education).mp. |
| #37 | mobilit* adj2 (train* or program*).mp. |
| #38 | mobilit* adj3 (aid* or device* or equipment*).mp. |
| #39 | walk* adj2 (train* or program*).mp. |
| #40 | hous* adj2 (adaptation* or accessib* or environment*).mp. |
| #41 | ambulat* adj2 (aid* or device*).mp. |
| #42 | 14 or 15 or 16 or 17 or 18 or 19 or 20 or 21 or 22 23 or 24 or 25 or 26 or 27 or 28 or 29 or 30 or 31 or 32 or 33 or 34 or 35 or 36 or 37 or 38 or 39 or 40 or 41 |
| #43 | exp Mobility Limitation/ |
| #44 | mobility status.mp. |
| #45 | exp Walking/ |
| #46 | walking.mp. |
| #47 | exp Gait/ |
| #48 | gait.mp. |
| #49 | exp Locomotion/ |
| #50 | locomotion.mp. |
| #51 | ambulat*.mp. |
| #52 | physical capacity.mp. |
| #53 | 43 or 44 or 45 or 46 or 47 or 48 or 49 or 50 or 51 or 52 |
| #54 | 13 and 42 and 53 |

Table S2: Search strategy for EMBASE database:

| #1 | exp Malignant Neoplasms |
| --- | --- |
| #2 | neoplasm*.mp. |
| #3 | cancer*.mp. |
| #4 | advanced cancer*.mp. |
| #5 | metastat* cancer*.mp. |
| #6 | incurable cancer*.mp. |
| #7 | terminal cancer*.mp. |
| #8 | tumo?r*.mp. |
| #9 | palliative cancer*.mp. |
| #10 | end stage cancer*.mp. |
| #11 | Stage 3 cancer*.mp. |
| #12 | Stage 4 cancer*.mp. |
| #13 | 1 or 2 or 3 or 4 or 5 or 6 or 7 or 8 or 9 or 10 or 11 or 12 |
| #14 | exp Rehabilitation Center/ or exp Rehabilitation Research/ or exp Rehabilitation/ |
| #15 | rehabilitation.mp. |
| #16 | exp Exercise/ or exp Aerobic Exercise/ |
| #17 | exercis*.mp. |
| #18 | activit* adj2 (motor or physical).mp. |
| #19 | exp Resistance Training/ |
| #20 | resistance train*.mp. |
| #21 | exp Patient Education/ |
| #22 | exp Health Education/ |
| #23 | exp Endurance Training/ |
| #24 | exp Daily Life Activity/ |
| #25 | endurance train*.mp. |
| #26 | exp Fitness/ |
| #27 | physical fitness.mp. |
| #28 | aerobic exercise*.mp. |
| #29 | exp Physiotherapy/ |
| #30 | physical therap*.mp. |
| #31 | physiotherap*.mp. |
| #32 | exp Occupational Therapy/ |
| #33 | occupational therap* |
| #34 | exp Electrotherapy/ |
| #35 | electrotherapy.mp. |
| #36 | gait adj2 (training or education).mp. |
| #37 | mobilit* adj2 (train* or program*).mp. |
| #38 | mobilit* adj3 (aid* or device* or equipment*).mp. |
| #39 | walk* adj2 (train* or program*).mp. |
| #40 | hous* adj2 (adaptation* or accessib* or environment*).mp. |
| #41 | ambulat* adj2 (aid* or device*).mp. |
| #42 | 14 or 15 or 16 or 17 or 18 or 19 or 20 or 21 or 22 23 or 24 or 25 or 26 or 27 or 28 or 29 or 30 or 31 or 32 or 33 or 34 or 35 or 36 or 37 or 38 or 39 or 40 or 41 |
| #43 | exp Limited Mobility/ or exp Patient Mobility/ or exp Physical Mobility/ |
| #44 | mobility status.mp. |
| #45 | exp Walking/ |
| #46 | walking.mp. |
| #47 | exp Gait/ |
| #48 | gait.mp. |
| #49 | exp Locomotion/ |
| #50 | locomotion.mp. |
| #51 | ambulat*.mp. |
| #52 | physical capacity.mp. |
| #53 | 43 or 44 or 45 or 46 or 47 or 48 or 49 or 50 or 51 or 52 |
| #54 | 13 and 42 and 53 |

Table S3: Search strategy for CINAHL database:

| S1 | (MH "Neoplasms+") |
| --- | --- |
| S2 | neoplasm* |
| S3 | cancer* |
| S4 | advanced cancer* |
| S5 | metastati* cancer* |
| S6 | incurable cancer* |
| S7 | terminal cancer* |
| S8 | tumo?r* |
| S9 | palliative cancer* |
| S10 | end stage cancer* |
| S11 | stage 4 cancer* |
| S12 | S1 OR S2 OR S3 OR S4 OR S5 OR S6 OR S7 OR S8 OR S9 OR S10 OR S11 |
| S13 | (MH "Rehabilitation+") OR (MH "Rehabilitation Centers+") OR (MH "Home Rehabilitation+") |
| S14 | rehabilitation |
| S15 | (MH "Exercise+") OR (MH "Resistance Training+") OR (MH "Therapeutic Exercise+") OR (MH "Group Exercise+") |
| S16 | exercis* |
| S17 | resistance train* |
| S18 | (MH "Patient Education+") |
| S19 | (MH "Health Education+") |
| S20 | (MH "Endurance Training+") |
| S21 | (MH "Activities of Daily Living+") |
| S22 | (MH "Physical Fitness+") |
| S23 | physical fitness |
| S24 | (MH "Aerobic Exercises+") |
| S25 | aerobic exercis* |
| S26 | (MH "Physical Therapy+") |
| S27 | physical therap* |
| S28 | physiotherap* |
| S29 | (MH "Occupational Therapy+") |
| S30 | occupational therap* |
| S31 | (MH "Electrotherapy+") |
| S32 | electrotherapy |
| S33 | gait n2 (training or education) |
| S34 | activit* N2 (motor or physical) |
| S35 | mobilit* N2 (train* or program*) |
| S36 | mobilit* N3 (aid* or device* or equipment*) |
| S37 | walk* N2 (train* or program*) |
| S38 | hous* N2 (adaptation* or accessib* or environment*) |
| S39 | ambulat* N2 (aid* or device*) |
| S40 | S13 OR S14 OR S15 OR S16 OR S17 OR S18 OR S19 OR S20 OR S21 OR S22 OR S23 OR S24 OR S25 OR S26 OR S27 OR S28 OR S29 OR S30 OR S31 OR S32 OR S33 OR S34 OR S35 OR S36 OR S37 OR S38 OR S39 |
| S41 | (MH "Physical Mobility+") |
| S42 | mobility status |
| S43 | (MH "Walking+") |
| S44 | walking |
| S45 | (MH "Gait+") |
| S46 | gait |
| S47 | (MH "Locomotion+") |
| S48 | locomotion |
| S49 | ambulat* |
| S50 | physical capacity |
| S51 | S41 OR S42 OR S43 OR S44 OR S45 OR S46 OR S47 OR S48 OR S49 |
| S52 | S12 AND S40 AND S51 |

Methodological quality assessments

Table S4: Quality appraisal of RCTs using JBI critical appraisal tool for assessment of risk of bias for RCTs

| First author/ year | Internal Validity | | | | | | | | | | Statistical Conclusion Validity | | |
| --- | --- | --- | --- | --- | --- | --- | --- | --- | --- | --- | --- | --- | --- |
|  | Was true randomisation used for assignment of participants to treatment groups? | Was allocation to treatment groups concealed? | Were treatment groups similar at the baseline? | Were participants blind to treatment assignment? | Were those delivering the treatment blind to treatment assignment? | Were treatment groups treated identically other than the intervention of interest? | Were outcome assessors blind to treatment assignment? | Were outcomes measured in the same way for treatment groups? | Were outcomes measured in a reliable way? | Was follow up complete and if not, were differences between groups in terms of their follow up adequately described and analysed? | Were participants analysed in the groups to which they were randomized? | Was appropriate statistical analysis used? | Was the trial design appropriate and any deviations from the standard RCT design accounted for in the conduct and analysis of the trial? |
| Bade, 2021 | Yes | Unclear | Yes | No | No | Yes | No | No | No | Yes | Yes | Yes | Yes |
| Cheville, 2013 | Yes | Unclear | Yes | No | No | Yes | No | Yes | No | Yes | Yes | Yes | Yes |
| Cheville, 2019 | Yes | Yes | Yes | No | No | Yes | Yes | Yes | Yes | Yes | Yes | Yes | Yes |
| Cormie, 2013 | Yes | Yes | Yes | No | No | Yes | No | Yes | Yes | Yes | Yes | Yes | Yes |
| Dhillon, 2017 | Yes | No | Yes | No | No | Yes | Unclear | Yes | Yes | Yes | Yes | Yes | Yes |
| Edbrooke, 2019 | Yes | Yes | Yes | No | No | Yes | Unclear | Yes | Yes | Yes | Yes | Yes | Yes |
| Galvão, 2018 | Yes | Yes | Yes | No | No | Yes | Unclear | Yes | Yes | Yes | Yes | Yes | Yes |
| Henke, 2014 | Yes | Unclear | No | No | No | Yes | Unclear | Yes | Yes | Unclear | No | Unclear | Unclear |
| Litterini, 2013 | Yes | No | Yes | No | No | Yes | Unclear | Yes | Unclear | Yes | Yes | Yes | Yes |
| Maddocks, 2009 | Yes | Yes | No | No | No | No | Unclear | Yes | Unclear | Yes | Yes | Yes | Yes |
| Maddocks, 2013 | Yes | Yes | Unclear | No | No | Yes | No | Yes | Unclear | Yes | No | Yes | Yes |
| Mendizabal-Gallastegui, 2023 | Yes | Unclear | No | No | No | Yes | Yes | Yes | Unclear | Yes | Yes | Yes | Yes |
| Mikkelsen, 2022 | Yes | Yes | Unclear | No | No | Yes | Yes | Yes | Unclear | Yes | No | Yes | Yes |
| Oldervoll, 2011 | Yes | Unclear | Yes | No | No | Yes | Unclear | Yes | Yes | Yes | Yes | Yes | Yes |
| Rutkoswska, 2019 | Yes | Yes | Yes | No | No | Yes | Yes | Yes | Unclear | Unclear | Unclear | Yes | Yes |
| Scott, 2018 | Yes | Unclear | Yes | No | No | Yes | Unclear | Yes | Unclear | Yes | Yes | Yes | Yes |
| Stuecher, 2019 | Yes | Yes | Yes | No | No | No | Unclear | Yes | Unclear | Yes | Yes | Yes | Yes |
| Uster, 2018 | Yes | Yes | Unclear | No | No | Yes | Yes | Yes | Yes | Yes | Yes | Yes | Yes |
| Vanderbyl, 2017 | Yes | Unclear | Yes | No | No | Yes | Yes | Yes | Unclear | Unclear | No | Yes | Unclear |
| Yee, 2019 | Yes | Yes | No | No | No | Yes | No | Yes | Unclear | Yes | Yes | Yes | Yes |
| Zimmer, 2018 | Yes | Unclear | Yes | No | No | Yes | No | Yes | Unclear | Yes | Yes | Yes | Yes |

Table S5: Quality appraisal of quasi-experimental studies using JBI critical appraisal tool checklist

| First author/ year | Is it clear in the study what is the ‘cause’ and what is the ‘effect’? | Were the participants included in any comparisons similar? | Were the participants included in any comparisons receiving similar treatment/care, other than the exposure or intervention of interest? | Was there a control group? | Were there multiple measurements of the outcome both pre and post the intervention/exposure? | Was follow up complete and if not, were differences between groups in terms of their follow up adequately described and analysed? | Were the outcomes of participants included in any comparisons measured in the same way? | Were outcomes measured in a reliable way? | Was appropriate statistical analysis used? |
| --- | --- | --- | --- | --- | --- | --- | --- | --- | --- |
| Avancini, 2023 | Yes | Yes | Yes | No | No | Yes | Yes | Yes | Yes |
| Avancini, 2024 | Yes | Yes | Yes | No | No | Yes | Yes | Yes | Yes |
| Chasen, 2013 | Yes | No | No | No | Yes | Yes | No | Yes | Yes |
| Cormie, 2014 | Yes | Yes | Unclear | No | No | Yes | Yes | Unclear | Yes |
| Delrieu, 2020 | Yes | No | No | No | Yes | Yes | No | Yes | Yes |
| Hanson, 2023 | Yes | Yes | Unclear | No | Yes | Yes | Yes | Yes | Yes |
| Kuerh, 2014 | Yes | Yes | Yes | No | Yes | Yes | No | Yes | Yes |
| O’Connor, 2020 | Yes | Yes | Unclear | No | No | Yes | Yes | Unclear | Unclear |
| Ozalevli, 2010 | Yes | Yes | Unclear | No | Yes | Yes | Yes | Yes | Yes |
| Park, 2019 | Yes | Yes | No | No | Yes | Yes | Yes | Yes | Yes |
| Quist, 2012 | Yes | Yes | Yes | No | No | Yes | Yes | Unclear | Yes |
| Quist, 2015 | Yes | Yes | Yes | No | No | Yes | Yes | Unclear | Yes |
| Schink, 2018 | Yes | Yes | Yes | Yes | No | Yes | No | Unclear | Yes |
| Schink, 2020 | Yes | No | No | Yes | Yes | Yes | No | Yes | Yes |
| Temel, 2009 | Yes | Yes | Yes | No | No | No | Yes | Unclear | Yes |
| Van Den Dungen, 2014 | Yes | Yes | Yes | No | No | No | Yes | Unclear | Yes |
| Zhao, 2016 | Yes | No | Yes | Yes | Yes | Yes | Yes | Unclear | Yes |
